# Recombinant human thrombopoietin improved platelet engraftment after autologous hematopoietic stem cell transplantation in patients with newly diagnosed multiple myeloma

**DOI:** 10.1002/cam4.4294

**Published:** 2021-09-26

**Authors:** Jingli Gu, Junru Liu, Xiaozhe Li, Waiyi Zou, Beihui Huang, Meilan Chen, Juan Li

**Affiliations:** ^1^ Division of Hematology The First Affiliated Hospital of Sun Yat‐sen University Guangzhou China

**Keywords:** clinical cancer research, multiple myeloma, survival

## Abstract

**Background:**

To evaluate the efficacy and safety of recombinant human thrombopoietin (rhTPO) for hematopoietic reconstitution after autologous stem cell transplant (ASCT) in patients with newly diagnosed multiple myeloma (NDMM).

**Method:**

Thirty‐five cases with NDMM had been enrolled into a prospective clinical trial from March 2014. The hematopoietic reconstitution was compared between these 35 cases (rhTPO group) and 98 historic cases not receiving rhTPO (control group) after stem cell reinfusion.

**Results:**

Thirty‐five (100%) cases receiving rhTPO achieved both neutrophil and platelet engraftment within 30 days post‐transplant. The median time to neutrophil and platelet engraftment was the 10^th^ day and 11^th^ day after stem cell reinfusion, respectively. Multivariate analysis showed that rhTPO administration was an independent factor for accelerating platelet engraftment (HR 2.013, 95% CI 1.336–3.034, *p* = 0.001). Subgroup analysis showed that rhTPO improved platelet engraftment and alleviated platelet transfusion needs in patients with inadequate re‐infused CD34^+^ cell counts of <2 × 10^9^/L. All the 35 patients tolerated rhTPO well. Survival analysis showed no decrease in time to progression (TTP) or overall survival (OS) by rhTPO administration.

**Conclusion:**

rhTPO accelerated the platelet engraftment after ASCT in patients with NDMM with good tolerability and long‐term safety, especially for those patients with poor CD34^+^ cell reinfusion. rhTPO might be recommended to be used early after ASCT for patients with NDMM.

## Graphical abstract

This study aimed to evaluate the efficacy and safety of recombinant human thrombopoietin (rhTPO) for hematopoietic reconstitution after autologous stem cell transplant (ASCT) in patients with newly diagnosed multiple myeloma (NDMM). Multivariate analysis showed that rhTPO administration was an independent factor for accelerating platelet engraftment. Survival analysis showed no decrease on TTP or OS by rhTPO application.

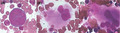

## 1 INTRODUCTION

Multiple myeloma (MM) is a malignant tumor of plasma cells.^1^ Autologous stem cell transplant (ASCT) remains the therapy of choice for young patients with newly diagnosed multiple myeloma (NDMM).^2^, ^3^ The high‐dose conditioning regimen of ASCT always causes myelosuppression which might lead to neutropenia and thrombocytopenia. Platelet engraftment could be delayed beyond 21 days after ASCT, in as high as 62% of patients receiving less than 2 × 10^6^/kg CD34^+^ cells reinfusion.^4^ For patients with sustained thrombocytopenia, bleeding increases the mortality and morbidity of ASCT,^5^ Hence, it is of clinical significance to explore methods to decrease the incidence of platelet engraftment delay and to accelerate the platelet engraftment.

Thrombopoietin (TPO) is an important growth factor regulating the platelet production.^6^ Recombinant human thrombopoietin (rhTPO), a full‐length and glycosylated TPO, had been applied successfully for patients with immune thrombocytopenia (ITP),^7^, ^8^ in stem cell mobilization for patients with MM,^9^ and for patients with solid tumors receiving non‐myeloablative chemotherapy.^10^ However, the role of rhTPO on platelet recovery in the context of hematopoietic stem cell transplantation is still controversial. Two phase I studies of rhTPO for patients receiving myeloblative chemotherapy in hematopoietic stem cell transplant showed no improvement on platelet recovery time.^11^, ^12^, ^13^ However, a prospective phase III study on the rhTPO for patients receiving haploidentical HSCT showed positive results on platelet engraftment after transplant.^14^

It is well recognized that the hematopoietic reconstitution is influenced by multiple factors including underlying hematological malignancies,^15^ stem cell sources,^16^ CD34^+^ cell counts,^16^ etc. To avoid the influence of different hematopoietic malignancies, our trial studied the role of rhTPO only in patients with NDMM and achieved at least partial response before transplant. Our study aimed to evaluate the efficacy and safety of rhTPO for hematopoietic reconstitution after ASCT in patients with NDMM.

## 2 METHODS

### 2.1 Patients and inclusion criteria

This single‐center, prospective phase II study was approved by the ethics committee of the first affiliated hospital of Sun Yat‐sen University and registered with the Chinese Clinical Trial Registry (ChiCTR1800017025). From March 2014 to March 2016, all consecutive eligible patients were enrolled. The eligibility criteria were as follows: (a) diagnosed with NDMM according to the IMWG criteria; (b) received <=8 cycles of induction regimen containing bortezomib; (c) achieved PR (partial response), VGPR (very good partial response), or CR (complete response) before transplant according to the IMWG criteria^17^; (d) planned to receive single ASCT; (e) not with dialysis‐dependent renal failure; (f) with normal heart function (EF>50%); (g) with bilirubin and AST/ALT <2 times of upper normal range; (h) with platelet counts ≥80 × 10^9^/L before conditioning; (i) had no history of allergy to rhTPO; (j) had not received melphalan or lenalidomide before ASCT; (k) not complicated with conditions leading to thrombocytopenia, such as ITP and MDS; and (l) not complicated with thromboembolism diseases or had new thromboembolism events within 6 months before conditioning. Eligible patients were enrolled before the conditioning and the written informed consents were obtained from patients in accordance with the Declaration of Helsinki.

The control group enrolled consecutively historic 98 NDMM patients since May 2007–April 2018. To minimize the characteristic difference between the rhTPO group and the control group, the control group patients also met the same eligibility criteria as the rhTPO group. Hence, all the control group patients were diagnosed with NDMM, achieved ≥PR response before transplant, not dialysis dependent, and not receiving any melphalan or lenalidomide in induction therapy. This retrospective part of the study was also approved by our ethics committee and the written informed consents were obtained from patients in accordance with the Declaration of Helsinki. Then the medical records were used to withdraw data on hematopoietic reconstitution until 90 days post‐transplant.

### 2.2 Treatments

For the rhTPO group, all patients received relatively unified treatments which had been described in detail in our previous report.^18^ Briefly, PAD (bortezomib, liposomal doxorubicin, and dexamethasone) induction was followed by subsequent ASCT. Conditioning regimen was high‐dose melphalan before 30 January 2015 and then changed to CBV regimen because of no supply of melphalan in China. Previous study had showed high‐dose melphalan and CBV conditioning had similar effects on platelet engraftment.^19^, ^20^ All patients underwent continuous maintenance with thalidomide/lenalidomide and/or interferon‐α.

### 2.3 Intervention

rhTPO (Sansheng Pharmaceutical Co., Ltd, Shenyang, China) was administered subcutaneously at a daily dose of 15,000 U for consecutively 14 days from day 1 or day 3 after stem cell reinfusion. The patients were randomly assigned to day 1 group or day 3 group at a 1:1 ratio using a table of random numbers. The rhTPO would be discontinued before schedule if platelet counts reached 100 × 10^9^/L or increased by more than 50 × 10^9^/L in a single day or severe side effects occurred. Single‐donor platelet with at least 2.5 × 10^11^ platelets was transfused at a platelet count of <20 × 10^9^/L or in the event of significant bleeding risk, regardless of the platelet count. Granulocyte colony‐stimulating factor (G‐CSF) was used when the patient had neutropenia until the neutrophil recovered to ≥0.5 × 10^9^/L. Complete blood counts were assessed at day −8, and then every day from the 1^st^ day post‐transplant to the day when the platelet recovered to ≥100 × 10^9^/L. If the patients could not achieve normal platelet counts beyond 30 days post‐transplant, complete blood counts were then tested every 7–14 days until 90 days post‐transplant. Renal, liver function, and electrolytes were monitored every 4 days from the 1^st^ day to the day when rhTPO was discontinued. Symptoms such as fever, headache, arthralgia, bone pain, fatigue, and dizziness were observed and recorded every day during rhTPO administration. At the 14^th^ day post‐transplant, bone marrow aspiration was performed for patients receiving rhTPO. Bone marrow megakaryocyte counts and differentiation were then analyzed. The surface markers of megakaryocyte were also analyzed by flow cytometry using antibodies against CD61, CD41, and CD42a.

### 2.4 Study endpoints and follow‐up

The study endpoints included (a) time to platelet engraftment which was defined as the first day of 7 consecutive days with a platelet count of ≥20 × 10^9^/L without transfusion support and (b) time to neutrophil engraftment which was defined as the first day of 3 consecutive days with a neutrophil count of ≥0.5 × 10^9^/L without the use of G‐CSF. For the comparison study between the rhTPO group and the control group, additional study endpoints included the platelet transfusion units, bone marrow (BM) smear at 1‐month post‐transplant, the time to progression (TTP), and overall survival (OS). The long‐term outcome results were reported as of 21 September 2019. The TTP was from the stem cell reinfusion to disease progression. The OS was from the stem cell reinfusion to death from any causes or the last day of follow‐up.

### 2.5 Statistical analysis

Chi‐squared (χ^2^) tests and *t*‐tests were performed for categorical and continuous variables fitting normal distribution, respectively. For continuous variates not fitting normal distribution, Mann–Whitney *U* tests were used. In the univariate analysis of the time to events, log‐rank tests were performed for the Kaplan–Meier survival analysis. In the multivariate analysis, the Cox proportional hazards regression model was used to adjust for significant variables of interest. The count of CD34^+^ cells infused was transformed into In (CD34^+^ cell counts) format before entered the Cox model. All statistical analyses were performed using SPSS 18.0 software. Values of *p *< 0.05 were considered statistically significant.

## 3 RESULTS

### 3.1 Patient selection and baseline characteristics

From March 2014 to March 2016, 35 consecutive patients with transplant‐eligible NDMM who also met the inclusion criteria of this study had been enrolled. The median age was 58 years (range, 34–69) for these 35 patients including 20 men (57.1%) and 15 women (42.9%). There were 16 (45.7%), 12 (34.3%), and 6 (17.1%) patients with international staging system (ISS) stages 1, 2, and 3, respectively. One case missed the ISS data. The patients received median 4 (range 2–7) cycles of induction chemotherapy containing bortezomib. Before the transplant, 45.7%, 37.1%, and 17.1% of cases had achieved response of CR, VGPR, or PR, respectively.

### 3.2 rhTPO facilitated platelet engraftment after ASCT in patients with NDMM

Of the 35 patients, 2 (5.7%), 16 (45.7%), 2 (5.7%), 13 (37.1%), and 2 (5.7%) cases received 10, 11, 12, 13, and 14 doses of rhTPO, respectively, with the median of 11 (range 10–14) doses of rhTPO delivered. Three (8.6%) patients stopped rhTPO before the 14^th^ day post‐transplant because they had already achieved platelet count >100 × 10^9^/L.

All 35 cases (100%) obtained neutrophil and platelet engraftment within 30 days post‐transplant. The median neutrophil and platelet engraftment time were the 10^th^ day (range 6^th^ to 20^th^) and the 11^th^ (range 8^th^ to 25^th^) day after the stem cell reinfusion, respectively. Thirty cases (85.7%) had normal platelet count ≥100 × 10^9^/L within 30 days post‐transplant, with the median time to PLT count ≥100 × 10^9^/L of the 17^th^ day (range: 14^th^ to 70^th^ day) post‐transplant.

To explore whether rhTPO could accelerate the hematopoietic reconstitution after ASCT in patients with NDMM, we compared the engraftment between the above 35 patients receiving rhTPO (rhTPO group) and historic 98 patients not receiving rhTPO in our center (control group). The age at diagnosis, ISS staging, conditioning regimen, and platelet counts before transplant had significant differences between the rhTPO group and the control group (Table 1).

**TABLE 1:** Baseline characteristics comparison between the rhTPO group and the control group.

	rhTPO group (n=35)	Control group (n=98)	*p* value
Sex, M/F, (n)	20/15	63/35	0.452
Age (yr), median(range)	58 (34–69)	52 (32–67)	0.028
Subtypes (n)			0.795
IgG	17	52	
IgA	6	21	
IgD	1	2	
Light chain only	11	23	
DS staging (n)			0.496
Ⅱ	1	8	
Ⅲ	34	90	
ISS staging (n)			0.036
1	16	24	
2	12	34	
3	6	39	
Missing	1	1	
Response before transplant (n)			0.876
CR	16	40	
VGPR	14	42	
PR	5	16	
Induction regimen			0.000
VD	0	34	
PAD	35	64	
Conditioning regimen (n)			0.001
HD‐Mel	15	75	
CVB	20	23	
Stem cell source (n)			0.780
BM	4	13	
PB	31	85	
PLT counts before transplant (10^9^/L)	184 ± 50	221 ± 105	0.008
TNC counts (10^8^/Kg)	4.00 ± 2.04	4.56 ± 2.24	0.195
CD34^+^ cell counts (10^6^/Kg)	3.48 ± 2.15	4.07 ± 3.34	0.331

DS, Durie‐Salmon; ISS, international staging system; CR, complete response; VGPR, very good partial response; PR, partial response; PLT, platelet; HD‐Mel, high‐dose melphalan; BM, bone marrow; PBSC, peripheral blood stem cell; TNC, total nucleated cell.

**FIGURE 1:**
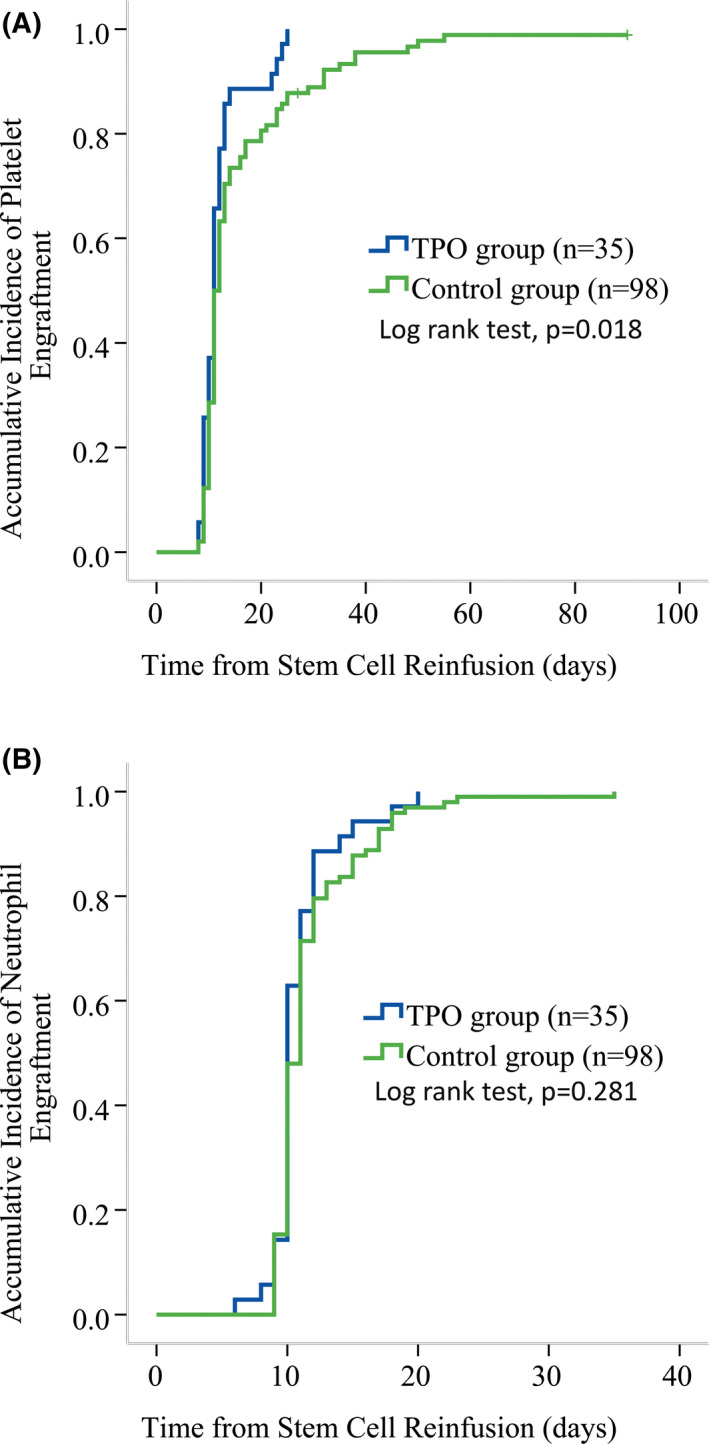
Comparison of platelet engraftment between patients of rhTPO group and of historic control group. Kaplan–Meier survival analysis showed that patients receiving rhTPO had quicker platelet engraftment than those not receiving rhTPO in the historic control group (A). However, the neutrophil engraftment was similar between the two groups (B).

In the univariate analysis, although the median time to platelet engraftment was the same of the 11^th^ day in the rhTPO group and the control group, the platelet engraftment incidence at the 14^th^ day and 28^th^ day post‐transplant was significantly higher for the rhTPO group than that for the control group (100% vs. 73.5 ± 4.5%; 100% vs. 87.8 ± 3.3%; log‐rank test, *p* = 0.018) (Figure 1A). No significant difference existed on the neutrophil engraftment between the rhTPO group and the control group with the median neutrophil engraftment time of the 10^th^ day and 11^th^ day post‐transplant, respectively (*p* = 0.281) (Figure 1B).

In the multivariate analysis including rhTPO administration (Yes vs. No), stem cell source (BMT vs. PBSC), In (CD34^+^ cell count), conditioning regimen (HD‐Mel vs. CVB), platelet counts before transplant, ISS staging, and age at diagnosis, rhTPO administration was the independent facilitating factor for the platelet (HR 2.013, 95% CI 1.336–3.034, *p* = 0.001) (Table 2).

**TABLE 2:** Multivariate analysis of factors influencing hematopoietic reconstitution.

Factors	Platelet engraftment time		Neutrophil engraftment time	
	HR (95%CI)	*p* value	HR (95%CI)	*p* value
Age	0.975 (0.957–0.994)	0.011	/	/
rhTPO (Yes vs. No)	2.013 (1.336–3.034)	0.001	1.459 (0.978–2.175)	0.064
In (CD34^+^ cell count)	2.204 (1.674–2.902)	<0.001	2.061 (1.573–2.699)	<0.001
Stem cell source (BMT vs. PBSCT)	0.271 (0.138–0.533)	<0.001	0.259 (0.129–0.519)	<0.001

BMT, bone marrow transplant; PBSCT, peripheral blood stem cell transplant.

To identify patients who might benefit the most from rhTPO administration, we did subgroup analysis according to the re‐infused CD34^+^ cell counts. The results showed that rhTPO improved platelet engraftment in patients with re‐infused CD34^+^ cell counts of <2.0 × 10^6^/Kg. For these patients with inadequate re‐infused CD34^+^ cells, the platelet transfusion need was also significantly alleviated in rhTPO group compared to the control group (median transfusion platelet of 3 vs. 6.5 units, *U* test, *p* = 0.044). For patients with adequate re‐infused CD34^+^ cell counts, there was no significant difference in the platelet engraftment time and platelet transfusion needs between the rhTPO group and the control group (Table 3).

**TABLE 3:** rhTPO administration improved platelet engraftment and reduced platelet transfusion needs in patients receiving inadequate CD34^+^ stem cell reinfusion.

Re‐infused CD34^+^ cell counts (10^6^/Kg)	N (%)			Median time to platelet engraftment (days post‐transplant)				Platelet transfusion units Median, (IQR)		
	rhTPO	Control	rhTPO		Control	HR (95%CI)	P	rhTPO	Control	p
<2	9 (25.7)	28 (28.6)	14		23	2.557 (1.126–5.804)	0.025	3 (1.5–6)	6.5(3–9)	0.044
≥2, <5	19 (54.3)	44 (44.9)	9		10	1.585 (0.908–2.766)	0.105	2 (1–2)	2 (1–2.75)	0.362
≥5	7 (20.0)	26 (26.5)	10		10	1.365 (0.582–3.194)	0.473	1 (1–2)	1 (1–2)	1.000
In total	35	98	11		11	1.526 (1.027–2.268)	0.036	2 (1–2)	2 (1–3.25)	0.119

IQR, inter‐quarter range, HR, hazard ratio.

### 3.3 rhTPO improved the megakaryocytopoiesis early after ASCT

**FIGURE 2:**
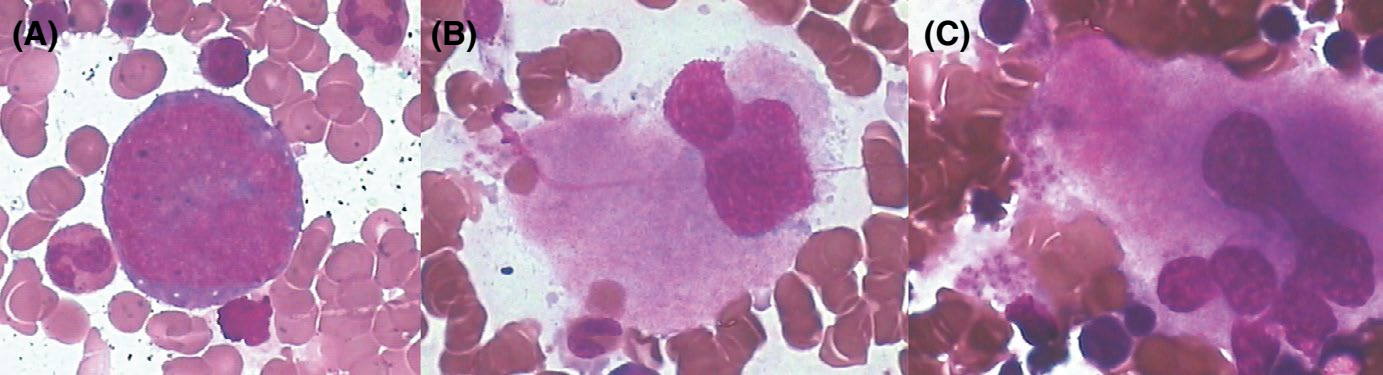
Megakaryocytopoiesis on the bone marrow smear of one patient receiving rhTPO early after transplant. (A) Megakaryoblast, (B) granular MK counts, and (C) thrombocytogenic MK.

Twenty‐two patients in the rhTPO group received bone marrow aspiration at the 14^th^ day post‐transplant to evaluate the bone marrow cellularity, proliferation, and maturation of megakaryocytes (Figure 2). The surface markers of megakaryocyte were also analyzed by flow cytometry using antibodies against CD61, CD41, and CD42a in 17 patients. Thirteen (59.1%), six (27.3%), two (9.1%), and one (4.5%) cases showed hyper‐cellularity, normal cellularity, hypo‐cellularity, and severe hypo‐cellularity on the bone marrow smear. The median counts of megakaryocyte (MK), megakaryoblast, granular MK, thrombocytogenic MK, and naked MK were 11 (0–100), 0 (0–8), 8 (0–80), 0 (0–21), and 0 (0–6), respectively. Twelve (54.5%) cases had normal MK count at the 14^th^ day after the stem cell reinfusion. The flow cytometry analysis of the bone marrow aspirate showed that the positive rate of CD61a, CD42, and CD41a was 0.37% (0.05–1.42%), 0.30% (0.01–1.34%), and 0.26% (0.02–0.96%), respectively. The total, granular, and thrombocytogenic MK counts at the 14^th^ day post‐transplant were positively associated with the platelet counts at the 14^th^ and 28^th^ day post‐transplant (Table 4). The expression of CD42 and CD41a was also correlated with the platelet counts at the 28^th^ day post‐transplant (Table 4).

**TABLE 4:** Correlation between platelet counts and the counts, and the surface marker expression of megakaryocyte in the BM at the 14^th^ day post‐transplant.

	Platelet at day 14		Platelet at day 28	
	r^a^	p value	r^a^	*p* value
MK counts	0.629	0.000	0.342	0.047
Megakaryoblast counts	0.426	0.011	0.133	0.452
Granular MK counts	0.615	0.000	0.361	0.036
Thrombocytogenic MK counts	0.651	0.000	0.405	0.018
CD61%	−0.066	0.753	0.201	0.335
CD42%	0.176	0.400	0.471	0.017
CD41a%	0.260	0.209	0.523	0.007

MK, megakaryocyte.

^a^ Because the variates were NOT of normal distribution, spearman's rho correlation statistics analysis was used accordingly.

Twenty‐two patients of the rhTPO group had both bone marrow smear at 14^th^ day and 1‐month post‐transplant. The total Mk cell counts (Δ counts of 5.22 ± 21.8, *p* = 0.264) and the thrombocytogenic Mk percentage (Δ percentage of 5 ± 15.7%, *p* = 0.144) did not differ between the BM at the 14^th^ day and that at 1‐month post‐transplant.

No control patient had undergone BM aspiration at 14^th^ day after transplant. However, bone marrow aspiration at 1 month was performed routinely in some control patients (n = 28) and most rhTPO patients (n = 28). Comparing the BM aspirate at 1 month between the rhTPO group and the control group, we found that the rhTPO group had higher Mk cell counts [16.5 (0–72) vs. 7 (0–100), *p* = 0.034], thrombocytogenic MK counts [2 (0–18) vs. 0 (0–15), *p* = 0.042], and thrombocytogenic MK percentage [1.21(0.00–0.38) % vs. 0.00 (0.00–0.24) %, *p* = 0.018].

### 3.4 Safety analysis and survival analysis

All the 35 patients tolerated rhTPO well and no patient discontinued rhTPO because of side effects. Low‐grade fever, fatigue, arthralgia, and somnolence were only seen in 1 (2.9%), 5 (14.3%), 1 (2.9%), and 3 (8.6%) cases, respectively. After rhTPO administration, only 2 (5.7%) and 3 (8.6%) cases had slightly elevated ALT levels which recovered to normal range after the discontinuation of rhTPO.

**FIGURE 3:**
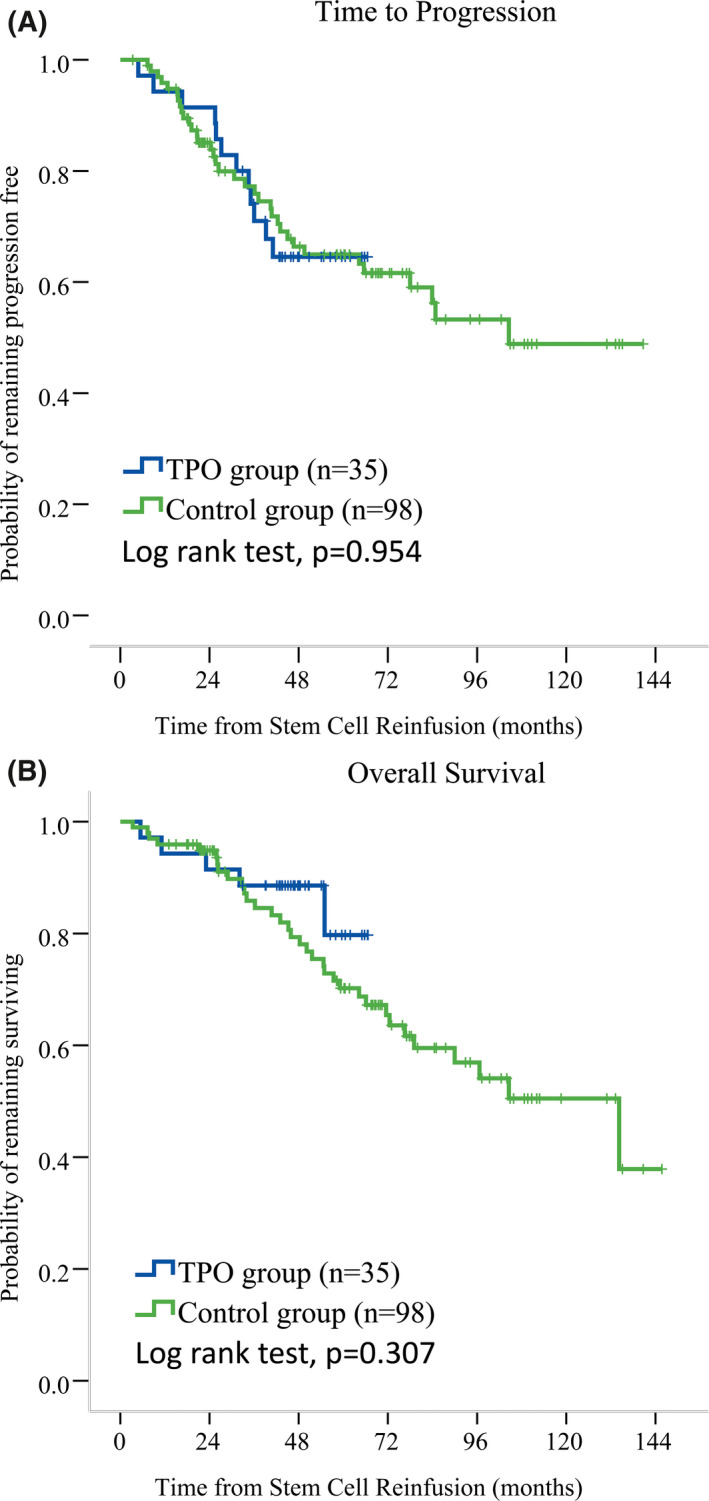
Long‐term outcomes comparison between the rhTPO group and the historic control group. Kaplan–Meier survival analysis showed that neither the median TTP (A) nor OS (B) was significantly different between the rhTPO group and the control group.

To determine whether rhTPO applied at the early post‐transplant period has the pro‐myeloma effects, we analyzed the long‐term survival for these 35 patients and compared their survival to that of the control group. The median follow‐up time for the rhTPO group was 47.8 (range 5.4–66.8) months. Neither the TTP nor the OS for patients of rhTPO group had been reached yet. Patients receiving rhTPO had similar median TTP (not reached vs. 104.6 months, *p* = 0.954) and OS (not reached vs. 134.2 ± 28.2 months, *p* = 0.307) to those of the control group (Figure 3).

## 4 Discussion

Multiple myeloma is the most common hematological malignancy to receive ASCT in the North America. Thrombocytopenia and neutropenia due to myelosuppression after ASCT might lead to severe spontaneous bleeding and infection which are the most common causes for transplant‐related mortality (TRM). Hence, new ways to facilitate hematopoietic reconstitution are needed to decrease TRM in these patients. However, no previous study has focused on the role of rhTPO for hematopoietic reconstitution after ASCT in patients with NDMM.

Our results showed that rhTPO could shorten the platelet engraftment time. However, two previous clinical trials of rhTPO for patients receiving high‐dose chemotherapy in ASCT showed no improvement on the platelet recovery or engraftment.^11^, ^12^ Why our result is different from the former studies? The first reason lied on the difference in rhTPO administration. The above two studies^11^, ^12^ only used rhTPO every 3 days for 10 doses. Instead, rhTPO was used daily for consecutively 14 days in our study and the study from Huang XJ group,^13^ both of which showed improvement effect of rhTPO on platelet recovery after HSCT. The second reason involved the underlying hematopoietic diseases before HSCT. The platelet engraftment after ASCT is significantly quicker in patients with MM or lymphoma than those with acute myeloid leukemia, with median platelet engraftment time of 12 versus 16–17 days.^14^ The earlier trials on rhTPO enrolled patients with heterogeneous underlying hematological malignancies^11^, ^12^ which might confound the true effects of rhTPO on platelet engraftment. Study from HXJ group focusing on leukemia showed positive effects of rhTPO on platelet engraftment.^13^ Similarly, our study only focused on single underlying malignancy of NDMM, on patients with unified induction regimen, and on relatively homogeneous disease status before ASCT. Hence, our trial was more powerful to detect the facilitating effects of rhTPO on platelet engraftment. Our study also showed that the role of rhTPO on improving platelet engraftment was more prominent in patients receiving inadequate re‐infused CD34^+^ cells, which suggested that rhTPO administration might be more beneficial for patients with risk of poor platelet engraftment.

Few studies reported megakaryocytopoiesis at as early as the 14^th^ day post‐transplant. Our results showed that about half patients had normal megakaryocytes counts at the 14^th^ day post‐transplant. However, the maturation of megakaryocytes was impaired because the megakaryocytes were dominated by granular megakaryocytes but not platelet‐producing megakaryocytes. The counts of granular megakaryocyte and platelet‐producing megakaryocytes correlated not only with the platelet counts at the 14^th^ day but also with those at the 28^th^ day post‐transplant. The expression of CD41a/CD42 indicating mature megakaryocytes also positively correlated with the platelet counts at 28^th^ day post‐transplant. The above results suggested that rhTPO had prolonged promotion effects on megakaryocyte maturation and platelet production, even the rhTPO administration was stopped at about 2 weeks post‐transplant.

No difference in the MK cell counts or maturation of MK cells was found between the BM at the 2^nd^ week and 1‐month post‐transplant. This longitudinal analysis indicated that the megakaryocytes reconstituted and matured within the first 2 weeks in patients receiving rhTPO early after stem cell transfusion, which supported the early use of rhTPO after the transplant. The rhTPO patient did have more MK counts and more mature MK cells than the control patients at 1‐month post‐transplant, which proved that rhTPO did have positive and prolonged impact on both the megakaryocyte production and maturation.

Recombinant human thrombopoietin was well tolerated in our enrolled patients. The symptoms of side effects were mild and no severe renal or liver function abnormalities were observed. These results suggested that rhTPO had good recent safety profile. In vitro study showed that TPO attributed to abnormal growth of leukemia cells.^21^ Although plasma cells usually do not express TPO receptor,^22^ study on animal model showed that the thrombopoietin pathway might bring to direct plasma cell expansion.^23^ Hence, it is necessary to explore whether rhTPO administration during early phase after ASCT would promote myeloma progression in the long term. Our results showed that patients receiving and not receiving rhTPO after ASCT had similar time to progression and overall survival, which indicated that rhTPO had no effects on the myeloma progression, hence had good long‐term safety profile.

Our study was a phase II clinical trial with small sample, phase III study is needed to further confirm the rhTPO facilitation effects on platelet engraftment after ASCT for patients with newly diagnosed MM.

## 5 Conclusion

Focusing on single hematological malignancy of NDMM, our study showed that rhTPO could improve the platelet engraftment, with good safety profile and without increased risk of tumor progression in the long‐term follow‐up. rhTPO might be recommended to be used early after ASCT for patients with NDMM, especially for patients receiving inadequate CD34^+^ stem cells reinfusion. Our study also enriched the understanding on the megakaryocytopoiesis after ASCT.

Future study was warranted to explore the efficacy of rhTPO on patients with risk of poor platelet engraftment. rhTPO might further be tried after ASCT for patients with other underlying hematological malignancies.

## CONFLICT OF INTEREST

The authors declare no conflict of interest in relation to the work described.

## AUTHOR CONTRIBUTION

Jingli Gu was responsible for design of the study, the data analysis, and the manuscript preparation; Junru Liu, Xiaozhe Li, Waiyi Zou, Beihui Huang, and Meilan Chen were responsible for the performance of the study; Juan Li was responsible for the design and performance of the study, and review of the manuscript and statistical analysis.

## ETHICAL APPROVAL

This study was registered at the Chinese Clinical Trial Registry with the registry number of “ChiCTR1800017025.”

All procedures performed in this study involving human participants were conducted in accordance with the ethical standards of the institutional and/or national research committee and with the 1964 Declaration of Helsinki and its later amendments or comparable ethical standards.

## Data Availability

The authors confirm that the data supporting the findings of this study are available within the article and its supplementary materials. Data will be sent to the public data bank of the first affiliated hospital of Sun Yat‐sen University and be available under request.
